# Synthesis and Characterization of Gold-Phosphine Complexes for Potential Use as Anti-Arthritic Agents

**DOI:** 10.1155/MBD.1994.518

**Published:** 1994

**Authors:** Francesco Caruso, J. Tanski, M. Rossi, C. Smart

**Affiliations:** 1 Vasar College Department of Chemistry Poughkeepsie NY 12601 USA; 2 Instituto di Strutturistica Chimica CNR, CP 10 Montemtondo Stazione Roma I - 00016 Italy


					SYNTHESIS
COMPLEXES
AND CHARACTERIZATION OF GOLD-PHOSPHINE
FOR POTENTIAL USE AS ANTI-ARTHRITIC AGENTS
Francesco Caruso,2, J. Tanski,M. Rossi and C. Smart
Vasar College, Department of Chemistry; Poughkeepsie, NY 12601, USA
2 Instituto di Strutturistica Chimica, CNR, CP 10, 00016 Montemtondo Stazione, Roma, Italy
New gold(I)-phosphine complexes have been synthesized and studied using multinuclear 1H. and
31p-NMR spectroscopy and X-my diffraction methods. 31p-NMR is particularly helpful as it
distinguishes between phosphorus atoms in different chemical environments. Gold(Ill) salts were
easily reduced in situ. A variety of diphosphines, L PPh2(CH2)nPPh2, n 1-4, and one
triphosphine, triphos, CH3C(CH2PPh2)3, were added stoichiometrically to the Au(I) solution so that
each gold atom was bound to one phosphorus.. These digonal gold complexes were isolated.
Monophosphines such as triphenylphosphine, PPh3, and tricyclohexylphosphine, PCy3, were added
in organic solvent to increase the metal coordination number. Several equilbria in solution will be
described together with diffraction studies performed on single crystals. In one case PCy3
displaced completely the diphosphine ligand.
518

				

